# Dignity in the care of older people – a review of the theoretical and empirical literature

**DOI:** 10.1186/1472-6955-7-11

**Published:** 2008-07-11

**Authors:** Ann Gallagher, Sarah Li, Paul Wainwright, Ian Rees Jones, Diana Lee

**Affiliations:** 1 Faculty of Health and Social Care Sciences, Kingston University & St George's University of London, Kingston Hill, KT2 7LB, UK; 2 Room 103, First Floor, Neuadd Ogwen, School of Social Sciences, Bangor University, Bangor, Gwynedd, LL57 2DG, UK; 3 Faculty of Medicine, Chinese University of Hong Kong, Shatin, N.T., Hong Kong

## Abstract

**Background:**

Dignity has become a central concern in UK health policy in relation to older and vulnerable people. The empirical and theoretical literature relating to dignity is extensive and as likely to confound and confuse as to clarify the meaning of dignity for nurses in practice. The aim of this paper is critically to examine the literature and to address the following questions: What does dignity mean? What promotes and diminishes dignity? And how might dignity be operationalised in the care of older people?

This paper critically reviews the theoretical and empirical literature relating to dignity and clarifies the meaning and implications of dignity in relation to the care of older people. If nurses are to provide dignified care clarification is an essential first step.

**Methods:**

This is a review article, critically examining papers reporting theoretical perspectives and empirical studies relating to dignity. The following databases were searched: Assia, BHI, CINAHL, Social Services Abstracts, IBSS, Web of Knowledge Social Sciences Citation Index and Arts & Humanities Citation Index and location of books a chapters in philosophy literature. An analytical approach was adopted to the publications reviewed, focusing on the objectives of the review.

**Results and discussion:**

We review a range of theoretical and empirical accounts of dignity and identify key dignity promoting factors evident in the literature, including staff attitudes and behaviour; environment; culture of care; and the performance of specific care activities. Although there is scope to learn more about cultural aspects of dignity we know a good deal about dignity in care in general terms.

**Conclusion:**

We argue that what is required is to provide sufficient support and education to help nurses understand dignity and adequate resources to operationalise dignity in their everyday practice. Using the themes identified from our review we offer proposals for the direction of future research.

## 1. Background

In United Kingdom health policy there is much rhetoric about dignity. Reports have highlighted ageism, care deficits and indignity in health and social care services [1-4]. Government responses have included, according to press reports, that every NHS hospital should have a 'dignity nurse' [5]. Reactions in the media were less than supportive of the initiative and it was described as "an insulting and cheap gimmick" [5]. The 'dignity nurse' proposal was abandoned in response to advice from senior nurses [6]. The policy documents and reports did not define dignity and the idea was used, for the most part, in a rhetorical manner and for dramatic effect. Nevertheless, the emphasis placed on dignity means that it cannot be ignored as an issue for health care professionals. Dignity in care is for example one of three themes in the report "A New Ambition for Old Age" [7], which outlines the next steps in implementing the National Service Framework for Older People and which should therefore be an influential document in the future planning and management of service for older people.

Dignity is not, of course, a new idea. Philosophically it can be traced at least as far back as the writings of Aristotle. It has an established place in human rights discourse and within, for example, the philosophy of the hospice movement. The first statement in the preamble to the 1948 Universal Declaration of Human Rights refers to "recognition of the inherent dignity and of the equal and inalienable rights of all members of the human family" .

In addition to increasing attention to indignity and policy responses, particularly in relation to the care of older people, there is a growing body of empirical and theoretical literature relating to dignity [8-16].

However, in spite of the wide-ranging body of literature relating to dignity, the common usage of the term seems more likely to confuse and confound than to clarify the meaning of dignity. If nurses are to be "personally accountable for actions and omissions", respecting the dignity of patients [17], clarification is an essential first step. To this end we critically examine three key questions relating to dignity: What does it mean? What promotes and diminishes dignity? How should it be operationalised in relation to the care of older people?

## 2. Methods

### 2.1 Dignity – Sources of Meaning

There are different approaches to understanding ideas or concepts such as dignity: we can think critically and philosophically about them; we can ask or observe people to find out what they understand by dignity, taking into account their experience and world view; or we can look to the humanities and consider accounts in novels, poetry, theatre or the visual arts. In this paper, we focus on the first two perspectives. Philosophers engage in critical reflection and offer typologies and accounts of dignity generally without reference to empirical data (other than from hypothetical examples, anecdote and personal experience). Social scientists collect, reflect on and derive themes, meanings and theories from empirical data from, for example, interviews and observation. These accounts are likely to be rich with emotion, experience and lived values.

The relationship between these disciplines and between theoretical and empirical perspectives on dignity is not straightforward and may most helpfully be viewed as a dialectical process, a conversation in which theory informs and generates empirical work and empirical work informs and challenges theory. In relation to dignity, a concept discussed and applied in relation to the everyday complexities of nursing practice, such a dialectic is necessary. Theory without empirical data is likely to be esoteric and disconnected from the reality of practice. Empirical data without theory enlightens neither the particular nor general aspects of practice and has the potential to lull the practitioner into unreflective positions of hopelessness or complacency.

### 2.2 Search strategy

We have not attempted to produce a systematic review, in the sense generally understood by this term. We have however attempted to be thorough and rigorous in our search for relevant publications and we therefore give a brief description of our search strategies. We adopted two approaches to locating relevant literature in relation to dignity: a conventional search strategy to locate empirical literature; and a broader approach enabling us to locate papers and book chapters in applied ethics and philosophy, enabling us to engage with a broad and historically wide-ranging body of literature.

Our search strategies for the empirical literature included a) a search of abstracts in Assia (Applied Social Sciences Index and Abstracts) and BHI (British Humanities Index); CINAHL (Cumulative Index to Nursing and Allied Health Literature), Social Services Abstracts, International Bibliography of the Social Sciences (IBSS), Wilsonweb (Social Sciences Fulltext and Humanities Fulltext), Web of Knowledge Social Sciences Citation Index and Arts & Humanities Citation Index, and b) hand-searched specialist journals. Our intention was to carry out a preliminary scoping of the potentially relevant literature so as to assess and ascertain the nature and distribution of relevant studies for breadth and depth. We excluded all review articles, discussion papers, anecdotes, non-English language studies and debates. Our inclusion criteria were primary empirical studies, older people and dignity, English language, quantitative and qualitative research. Our search covered all the dates from 1951 to April Week 02, 2007. We used the following key words: dignity, older, geriatric, gerontology, aging/ageing, senior citizen, OAP/OAPs, pensioner, old or elderly or elder or elders.

From a total of 342 abstracts, we identified 49 empirical studies which explored the concept of dignity within the context of health and social care settings for older people. Papers were selected on the basis of their potential to display representative features of dignity.

The approach we employed to engage with philosophical perspectives on dignity included electronic and hand searches of philosophy and applied ethics journals and a scrutiny of philosophy texts and chapters. Dignity is a well-established concept in Western philosophy and some of the writings predate electronic search strategies, for example, Aristotle (384-322 BC) and in the 18^th ^Century, Immanuel Kant. We also identified readings from bibliographies and reference lists in papers relating to concept analysis.

## 3. What does dignity mean? The philosophy literature

It seems generally to be accepted that the concept of dignity means something like being of value or worth, because of the presence of some necessary characteristics. One of the earliest references to dignity is in Aristotle's Eudemian Ethics [18] where it appears as one of fourteen virtues or mean states of character between an excess of unaccommodatingness and of deficiency or servility [18]. Dignity for Aristotle is thus a quality, an excellence or moral virtue of the person, a quality that contributes to human flourishing or happiness and one in which one can err in terms of excess or deficiency. If an individual has too little sense of her own worth she may be servile and if too much she may not accommodate others and may be guilty of the vice of arrogance.

A more recent account of dignity comes from the 18^th ^Century philosopher, Immanuel Kant, who argues that some things have a price for which they can be exchanged or for which their value can be traded, but some things are beyond price and cannot be exchanged. For Kant these have worth or dignity [19]. As Badcott argues, Kant holds that human beings posses dignity because "they are rational, autonomous creatures with intrinsic value who can pursue and determine their own ends" [20]. For both Aristotle and Kant dignity thus seems to be contingent upon characteristics such as rationality and autonomy: it would be difficult for someone who lacked rationality to possess the Aristotelian moral virtues, while Kant's reference to "intrinsic value" seems nonetheless to rest on the possession of autonomy.

Some contemporary philosophical accounts also emphasise individual capability or autonomy in relation to dignity. Shotton and Seedhouse [13], for example, define dignity in relation to the interplay between capabilities and circumstances, pointing out that "we tend to lack dignity when we find ourselves in inappropriate circumstances, when we are in situations where we feel foolish, incompetent, inadequate or unusually vulnerable". They hold that dignity can be maintained where there are the capabilities to respond to potentially undignifying circumstances or where the circumstances are changed so they are not undignifying. If, for example, an older person felt that wearing an open-backed hospital gown was undignified the person could either ask for an alternative, or nurses could, as occurred in one of our local Trusts, redesign the gown so it opened at the side rather than the back. Elsewhere Seedhouse argues that "if a health worker wants to promote a person's dignity she must either expand her capabilities or improve her circumstances" [21]. This perspective focuses on whether a person feels dignified or undignified, rather than on whether others perceive them as having dignity, thus making dignity a subjective experience rather than a moral quality subject to the judgement of others.

Pullman [22] distinguishes between an ethic of dignity and an ethics (*sic*) of autonomy in relation to long-term care. He points out that autonomy "is crucial to certain aspects of dignity, but should not be confused with the whole of it" [22]. This discussion supports the inadequacy of autonomy as the sole ethical focus of care, particularly in relation to those who lack autonomy. Pullman's view of an ethic of dignity does not, however, deny the importance of autonomy as a value and he states that:

each autonomous citizen assumes some paternalistic responsibilities to protect the dignity of others who may never have the capacity, are not yet capable, or who are no longer able, to care for themselves – recognises and values our mutual interdependence. It is respect for the basic dignity of humanity that elicits our care and concern for the severely demented and frail older person. In responding to their dignity we express and enhance our dignity as well.

While Pullman recognises the importance of autonomy, he emphasises its limitations as a value ("it is a value, not the value") and suggests the importance of dignity, particularly where autonomy is lacking.

Beyleveld and Brownsword [23] develop the relationship between autonomy and dignity further and demonstrate the tension that may exist between these two concepts. They discuss dignity and the conditions in which human rights can flourish. Where a person is autonomous dignity can, they argue, be a "two-edged sword". It can either empower and support dignity or constrain it. To illustrate this, Beyleveld and Brownsword give the example of a French response to the activity of dwarf-throwing [23]. The Council d'État affirmed that respect for human dignity was one of the components of *ordre public *and, therefore, the so-called attraction of dwarf-throwing in local clubs should be banned. One of the dwarfs involved, Manuel Wackenheim, argued that he freely participated in the activity, that it secured him a monthly wage and enabled him to engage in professional life. The Council d'État responded that Wackenheim "compromised his own dignity by allowing himself to be used as a projectile, as a mere thing, and that no such concession could be allowed" [23]. We return to this discussion of the relationship between autonomy and dignity in the concluding sections of this paper. Jacelon [24] brings these concepts together, relating dignity to integrity. She describes personal integrity as "a dynamic intrinsic quality of the self, composed of health, autonomy and dignity".

A range of types or categories of dignity appear in the literature. Sandman [25], for example, refers to human dignity and contingent dignity. Badcott [20] writes of emotional dignity and distinguishes between intrinsic and extrinsic dignity: the former something that everyone has just because they are human and the latter contingent or extrinsic. Mann [26] distinguishes between internal (how I see myself) and external (how others see me) components of dignity. Spiegelberg [27] distinguishes between: the expression of dignity by inward and outward behaviour; dignity in general (a matter of degree); human dignity (minimum dignity which belongs to every human being qua human); intrinsic and extrinsic dignity; relational and absolute dignity; and dignity in itself (intrinsic worth) and ground for dignity and worthiness of respect.

Two theoretical accounts are particularly helpful in identifying features of dignity and indignity in relation to health and social care. The first, accommodating both intrinsic and extrinsic or contingent features, is that by Nordenfelt [28,29] (for a fuller account of Nordenfelt's approach see Wainwright & Gallagher 2008). This framework provided the philosophical backdrop to the Dignity and Older Europeans Project [15]. Nordenfelt distinguishes between intrinsic and contingent value, but he divides the latter into three, and thus distinguishes four concepts or varieties of dignity as follows:

• *The dignity of Menschenwürde *– Menschenwürde meaning an intrinsic dignity we all have to the same degree just because we are humans.

• *Dignity of merit *– People have rights on the basis of holding certain roles or office or because they have earned merit through their actions. They have rights on the basis of merit and are, therefore, treated as having a special dignity.

• *The dignity of moral stature *– This kind of dignity is based on their moral stature that emerges from their actions and omissions and from the kind of people they are. There are degrees of this and it is dependent on subject's action so may come and go.

• *The dignity of personal identity *– This kind of dignity is related to one's identity as a person and is related to self respect and concepts such as integrity, autonomy and inclusion. This kind of dignity can be taken away from people when, for example, they are humiliated, insulted or treated as objects.

The concept of Menschenwürde is of particular relevance to nursing practice as it emphasises the importance of acknowledging the worth of all human beings, regardless of their condition and is thus a counter to the criticisms of rationalist models of dignity described above. Dignity of merit and dignity of moral stature are interesting from a nursing practice perspective. Clearly, for Nordenfelt, people who attain high office or who demonstrate great moral probity deserve respect on that account and, if for no other reason than common courtesy nurses should give all patients appropriate respect. However an appeal to Menschenwürde could be said to override any claim to particular respect for merit or moral stature in so far as nursing practice is concerned. Health care professionals are generally expected to treat all patients who come before them, regardless of their moral character or civic status. Given the view of Aristotle that dignity is one of the moral virtues the moral account of dignity is clearly important. Aristotelian virtue theory also reminds us of the importance of dignity as a quality of the health care professional. Nurses, by this account, would be expected not only to respect the dignity of patients but also to exhibit dignity in their own character. Dignity of identity is also of particular interest to nurses, as it has the potential to give the clearest guidance as to how we should treat other people in practice, so as to preserve their dignity. The importance of dignity of identity provides, for example, a theoretical justification for providing individualised care.

While Nordenfelt [28,29] identifies four positive types or varieties of dignity, Mann [26] developed a provisional taxonomy of dignity violations as follows:

• *Not being seen *– This occurs when someone feels that they are not acknowledged or recognised and where people feel unheard or disregarded. Mann suggests that an extreme example is where prison and concentration guards were instructed not to make eye contact with inmates and to 'look only at the centre of their forehead'. An example from nursing practice might be the patient or visitor who tries to attract the attention of a nurse, only to have the nurse avoid eye contact and to ignore the attempt at engagement.

• *Being seen but only as a member of a group *– In such situations people may be seen but only as a stereotypical member of a group, for example, as a woman, student, Italian, older person or a schizophrenic. As Mann [26] points out, ''group classification can be a source of pride'' but here, as a type of dignity violation, being seen only as a group member is pejorative and depersonalising, diminishing the dignity of the individual.

• *Injuries to dignity resulting from violations of personal space *– There are differences in the way we perceive personal space and how we respond to people who enter our personal space. Responses will vary according to the nature of the relationship, whether permission has been gained and how dignified people feel when someone enters their personal space. There is much potential for dignity violations should permission not be sought and gained.

• *Humiliation *– This final type of dignity violation may occur if people are singled out, separated or distinguished from the group and subject to criticism. Mann gives the example of a child who is asked to stand in the corner at school. Although Mann refers to the conscious ''singling out'' of an individual, humiliation may equally follow from not being recognised as an individual, as in each of the previous three categories. Thus, although Mann calls this a dignity violation it might also be seen as the result of any other dignity violation. If we are not seen or seen only as a member of a group, or if our personal space is violated and we are thus treated as being of little worth, humiliation would describe our affective response to the experience and might also characterise how others would describe our situation.

Mann's provisional taxonomy was informed by discussions with students, anthropologists, sociologists and bioethicists. What becomes clear from the discussion of theoretical or philosophical perspectives on dignity is the necessary engagement of philosophy with empirical data about human experience and with the work of the social or human sciences. This is an example perhaps of Bhaskar's description of philosophy as underlabouring, a role it plays "for the sciences, and especially the human sciences, in so far as they might illuminate and empower the project of human self-emancipation" [3].

## 4. Dignity as a nursing value

Nurses and other health care professionals are frequently exhorted to respect the dignity of patients and clients. Respect for dignity appears as a central value within nursing codes. The preamble to the International Council of Nursing Code [31] states:

Inherent in nursing is respect for human rights, including cultural rights, the right to life and choice, to dignity and to be treated with respect.

The Code for nurses in the United Kingdom [17] states :

Make the care of people your first concern, treating them as individuals and respecting their dignity.

There is, then, agreement within nursing codes that respect for dignity is an important value and that nurses have obligations to respect the dignity of patients. However, what this requires is not made explicit and there is no agreement that dignity is a necessary component of ethical healthcare practice.

It has been argued that the application of the concept of dignity is lacking in normative or explanatory value. Mann, discussing the Universal Declaration of Human Rights complains that "the UDHR is largely silent about the meaning or implication of dignity" [26]. Similarly, Schulman [32] points out that such declarations do "not offer clear and unambiguous guidance on bioethical controversies". The Department of Health [33,34] website notes that (in spite of all the Government rhetoric about the importance of dignity), "There is no clarity about what dignity is and what minimum standards for dignity should be"; in spite of this DH states that it is their aim "to create a zero tolerance of lack of dignity in the care of older people". Sandman [25] is sceptical about the usefulness of the concept of dignity in relation to palliative care, arguing that it is "difficult to see that we deserve, owe or are owed anything just for being human", but that it is also difficult to find other criteria for human dignity that are sufficiently inclusive to accommodate all people as having equal worth, while excluding non-human animals. For Sandman it is "far from obvious that we have any use for the concept of human dignity" in nursing care.

Another criticism of dignity as a concept in healthcare ethics comes from Macklin, who states that "Appeals to human dignity populate the landscape of medical ethics" [35] and points to references to dignity in human rights declarations and bioethics reports. She argued that dignity is a "useless concept in medical ethics and can be eliminated without any loss of content". She goes on to say that "in the absence of criteria that can enable us to know just when dignity is violated, the concept remains hopelessly vague" and that it "means no more than respect for persons or their autonomy". Macklin's paper in the British Medical Journal in 2003 generated responses arguing for and against the utility of dignity . If Macklin is correct in saying that dignity equates with respect for persons or their autonomy then it seems plausible that the additional terminology of dignity is not necessary and we should simply demand that people are treated with respect. Without an analysis of respect [36] this is less than helpful. However, from our review of the literature and interdisciplinary discussions, we would argue that dignity is fundamentally concerned with claims of worth or value, with behaviour that justifies such claims and with treatment by others that shows appropriate respect: dignity is thus not reducible merely to autonomy or to respect. Further, the negative implications of dignity can be avoided and it serves an important function in nursing ethics and is then a necessary and appropriate nursing value.

## 5. What promotes and diminishes dignity in practice? Learning from empirical findings

A dialectical relationship between theoretical analysis and empirical studies in relation to dignity, and the underlabouring role of philosophy, is, arguably, particularly important in relation to the care of older people. In discussing how sociological theory informs their empirical study, Calnan et al [9] argue that:

Theoretical accounts have offered a general understanding of the social significance and importance of dignity and suggest that older age may threaten dignity by structuring and limiting the opportunities for participation and/or social recognition. Micro-sociological research has shown how older people negotiate their identity, in the face of its erosion by the aging body and disability and the domination of health and social care workers.

Any plausible account, therefore, of what promotes and diminishes dignity in practice should be grounded in theoretical accounts of dignity and ageing from philosophy and sociology, together with the analysis of empirical data. This might involve, for example, exploring the conceptual relationship between dignity and other values, such as autonomy and respect; the varieties or types of dignity and dignity violations and the way in which older people construct their identities and experience dignity in their lives.

Empirical studies of dignity have investigated the views of older people in nursing homes [1] and the views of hospitalized older people [12,37,38]. The most comprehensive European study of older people and dignity, which resulted in a large number of publications, theoretical and empirical, was led by Tadd and colleagues [8,14,15,39-41]. This study obtained the views of older people, young and middle aged people and health and social care professionals. The majority of the studies we located were European or American. One exception was the work of Lee and colleagues [42] in Hong Kong where the views of older people were obtained regarding their views of privacy and dignity and what supported or undermined these values. This work is ongoing and is being replicated in the UK. The empirical studies are all qualitative in nature and the methods of data collection include interviews, focus groups and observation.

In one of the publications relating to the Dignity and Older Europeans study [39], it was reported that older people in the United Kingdom viewed dignity as a multi-faceted concept with the following components: dignity of identity; human rights and autonomy. The data suggests how each of the components can be maintained or compromised by the behaviour of the person themselves, the behaviour of staff and by the environment. In relation to dignity of identity, for example, there is reference to 'they let themselves go' and to staff referring to older people in a derogatory way, for example, as 'cotton buds', 'wrinklies' or 'geriatrics'. Mixed sex wards were considered undignifying. In relation to human rights, examples are given of the right to choose in relation to end of life care and to rights in terms of adequate pensions. In relation to autonomy, there is emphasis on independence and control over one's life. Professionals' views of dignified care within the European study [41] shared the themes of autonomy and maintenance of identity and also included: a holistic and person-centred approach; participation, communication and respect. Professional views of undignified care have similarities with the dignity violations outlined by Mann [26], that is, invisibility, depersonalized care, treatment as an object, humiliation and abuse and mechanistic approaches to care. Another paper from the study [8], reporting the perspectives of those who work in health and social care, highlighted the importance of identity, human rights and autonomy and pointed to challenges regarding resources and a task oriented approach. A gap was identified between what providers are able to deliver and what they would like to deliver. Differences were noted in time frames between older people and staff – the former being time-rich and the latter time-scarce.

A Swedish study [10] suggested three themes that illustrate positive and negative aspects of ageing and vulnerability in relation to dignity. They were: the unrecognizable body; fragility and dependence; and inner strength and a sense of coherence. Empirical data from the studies cited provide rich perspectives from older people on their views and experience of dignity and indignity and suggest factors or components that contribute to or detract from dignity in practice.

There are many similarities among the various empirical studies. Findings from the 'Dignity and Older Europeans' study and from the Seedhouse and Gallagher [12,38] study supported the significance of: staff behaviour and attitudes; the environment and culture of care; and resources. These themes are also apparent in the recent Department of Health Survey, which obtained the views of professionals and members of the public over a ten week period (from June to September 2006) regarding dignity in care. The findings were published on the Department of Health web-site. . The survey generated over 400 responses, 240 from healthcare professionals and the remainder from members of the public. The DH survey is interesting, not because it is necessarily rigorous research – we know little about how it was analysed, the nature of the (self-selected) sample, or the implications of internet access, for example – but because it represents a kind of official account and has been presented as the background to future policy work and to exhortations to the professions to do better. Reports relating to the survey published [33,34] outlined ten of the 'most commonly raised issues in the survey' and two 'minor issues' (ibid p.5). The ten most common issues are as follows:

1. Clarifying what dignity is – findings suggested that there is no clarity about what dignity is and what minimum standards should be. Responses suggested a range of meanings, for example, privacy, courteous treatment, having choices about care and consideration for cultural and religious needs.

2. Complaining about services – it was reported that 'the overwhelming majority of people who completed the survey' felt that It is difficult to make a complaint about services, that the complaints system is not adequate and needs to be more accessible, simpler, quicker to respond, more independent and more powerful.

3. Being treated as an individual – responses suggested that people were not listened to or treated as an individual and that they were being cared for as a group. Suggestions for good practice included: talking to people as individuals and not stereotyping them; encouraging independence and giving people time and choice.

4. Privacy in care – People reported not having enough privacy when receiving care. The environment is important here ensuring that curtains and private rooms available and also protecting privacy of information.

5. Assistance in eating meals – It was reported that there is not enough assistance available or time allocated to service users to eat meals.

6. Access to lavatory/bathroom facilities – There is often insufficient access to lavatory/bathroom facilities with staff unavailable to help and alternatives, such as commodes, offered that people found embarrassing and undignified.

7. Being addressed by care staff appropriately – Responses emphasised the importance of using proper titles and not calling people 'love', 'dear', 'poppet' and so on.

8. Maintaining a respectable appearance – Lack of care, time and resources and laundry damage were said to contribute to people not appearing well-groomed.

9. Stimulation and a sense of purpose – it was felt that lack of stimulation can speed decline and make people feel isolated, therefore, having stimulating activities and a sense of purpose (when in a care home or at home alone) are important.

10. Advocacy services – People suggested that there are insufficient advocacy services for vulnerable adults and that these would support people in making complaints.

The two other issues that were identified as "common issues" and in relation to which there were "a smaller number of comments about" were labelled "minor issues". This appears to be unfortunate and inappropriate terminology given the potential of these issues to diminish dignity for service users, as the two items were:

1. Language barriers between care staff and service users – Responses pointed to difficulties in communication and cultural differences in care.

2. Mixed-sex facilities – Being placed in mixed-sex facilities makes many people feel uncomfortable

The NHS depends on a large number of staff from outside the UK, for whom English is not their first language, while the patient population also represents considerable ethnic diversity. Language barriers have the potential to create problems and an inability to communicate effectively may lead to problems for the maintenance of dignity. Placing patients in mixed-sex facilities may only have generated a smaller number of comments but it can hardly be dismissed as a minor issue, given the amount of attention paid to it by government ministers and political parties and in the media in recent years, an issue we return to later in this paper.

## 6. Dignity themes

The examples of poor care identified from the Department of Health data are graphic and are not dissimilar to examples cited elsewhere. However they are too specific to be useful in a more general consideration of dignity in care, for which we would argue a thematic analysis is more helpful. We compared the Department of Health [33] findings to earlier empirical work and to theoretical frameworks. Following an analysis of both we concluded that concerns about dignity could be organised in four common themes:

• environment of care;

• staff attitudes and behaviour;

• culture of care; and

• specific care activities.

The first theme, *environment of care*, sets up the context in which care is given and the conditions which may lead to patients not being treated as being of worth. The environment of care, and most particularly the physical environment, includes issues of privacy and of the nature of the institution. This theme is evident in previous empirical work, as discussed above. In the Department of Health report four of the twelve issues identified related to the environment of care. Privacy in care, access to lavatory/bathroom facilities, stimulation and a sense of purpose and mixed sex wards all have the potential to impact on patient dignity

As a social convention, if we feel that the users of a facility are important people we take some trouble to ensure the accommodation is of good quality. An unsatisfactory environment of care thus implies a failure to recognise the worth or value of the patient or service user. Where there are gaps in curtains, lack of privacy for examinations, insufficient access to toilets and bathrooms, mixed sex wards, and drab and shabby accomodation both basic human dignity or Menschenwürde and dignity of identity [28,29] are compromised. Violations of personal space and humiliation, as outlined by Mann [26], are also potential dignity violations.

*Staff attitudes and behaviour: *this theme concerns factors reported by respondents in several studies that reflect the way individuals responded to patients, showing a lack of respect, intolerance, impatience, and being patronising. Staff attitudes and behaviour included infantilising and patronising approaches, respondents to the DH survey saying for example, "the use of endearments such as sweetheart, darling, poppet should be banned from health care language. These terms are predominantly used in communicating with older people and it is inappropriate, demeaning and patronising". Patients felt that care that contributed to dignity required appropriate use of language, empathy, kindness and showed that the nurse knew the patient as an individual.

This was a strong theme in other reports of empirical work we examined. In the Dignity and Older Europeans Project [16] older people emphasised the importance of carers and others showing respect and recognition. This theme is also evident in at least four of the issues identified in the Department of Health [34] report: being treated as an individual; being addressed by care staff appropriately; maintaining a respectable appearance; and language barriers between care staff and service users. Staff attitudes and behaviour have the potential to enhance dignity in these areas when care is individualised and people not stereotyped, when appropriate terms of address are agreed, when time and care is invested in helping people to dress and be groomed as they see fit; and when communication is improved to facilitate collaborative and patient-centred care.

From a theoretical perspective concerns in this area would seem to reflect Menschenwürde and dignity of identity. By the accounts of Nordenfelt and others, we should treat the unconscious, demented or confused patient with as much respect, tolerance, patience and empathy as we would any other person, because such patients remain human beings with human dignity. Dignity of identity and self respect are violated by behaviour that is disrespectful of dignity because such behaviour results in low self esteem, loss of self respect and feelings of lack of worth. This also resonates with dignity violations outlined by Mann [26]: not being seen, being seen but only as a member of a group and humiliation.

*The culture of care *indicates factors that suggest in general the shared beliefs and values concerning the nature, style and organisation of care that may prevail in an area. This is related to what is often called the "Ward Philosophy", although we prefer the term "culture" to capture the sense of shared beliefs and values. Thus respondents wished for, but were often denied, the opportunity to be involved in their care, to express their autonomy, to be allowed to give or withhold consent, and to be treated as individuals, in an atmosphere that respected cultural differences and offered confidentiality. Having accessible and transparent complaint processes, often denied according to the Department of Health report is also suggestive of the environment of care. If the culture of care is positive rather than defensive and focused on therapeutic goals and patient/service-user well-being then complaints processes will be views in constructive terms. Similarly the availability of advocacy services, an improvement suggested by the Department of Health survey [33], would be viewed favourably.

Concerns regarding the culture of care seem particularly to reflect dignity of identity, as these are the concerns of the autonomous, rational individual seeking to preserve self respect and self-identity. They are underpinned by concern for human dignity, as this provides the basis for efforts to involve patients as much as possible even when this is difficult, and to provide individualised care wherever possible even if this has to be inferred from secondary information, about for example a patient suffering from advanced dementia. Budgetary constraints, a concern with performance targets, prioritising the institutional objectives over the needs of patients, trying to discharge patients as quickly as possible and staffing arrangements that result in many different nurses caring for the patient over any given period were all cited by respondents as examples of care that lacked dignity. The theme of the culture of care is supported by references to holistic and individualised care and to participation, to the failure to provide holistic and individualised care relates to menschenwürde and to dignity of identity. In this theme we get a sense of an organisational climate in which the institution's goals take priority over the objectives of practice.

The fourth and final theme relates to the wide range of *specific care activities *that have the potential to promote or thwart dignity, for example, to actual procedures or actions, such as bathing, toileting, feeding, dressing and so on. Respondents to the DH survey [33] mentioned these frequently as examples of undignified care, describing patients being left in soiled beds or clothing, not given help with meals or drinks, not being dressed appropriately, or being placed in situations where privacy was ignored, and similar concerns have been raised in many other reports, for example in the media. Such indignities by definition would be inflicted on the most dependent patients, often on those who lacked capacity through dementia. This theme relates particularly to the DH [33] issues relating to assistance in eating meals, privacy and access to lavatory and bathroom facilities.

Attention to small details of care and to individual preferences in relation to care activities are highlighted as being of much significance and suggests how the different themes may be interdependent. Gallagher [44], for example, describes the preference of an older female patient for a cup and saucer. To young people accustomed to drinking from a mug or a Styrofoam cup from a coffee shop this may seem foolishness, but to a woman of a certain generation and social class, who would never dream of using a cup without a saucer, and would be ashamed to serve tea to a guest in this way, this would be a significant matter. If not providing a saucer was just laziness or thoughtlessness on the part of the nurse then the responsibility is hers. But if the institution has chosen, perhaps as some cost control measure, to remove crockery from the ward and to serve all drinks in plastic cups from dispensing machines, then the disrespect is institutionalised and nurses will have great difficulty overcoming this. In this context the first appeal would be to Menschenwürde, as depriving conscious or unconscious patients of adequate privacy or care for hygiene, nutrition, or elimination seems a straightforward violation of human dignity. Attention to the nuances and preferences of individual patients also points to the importance of dignity of identity and to nursing responses that engage with patients in their care.

What becomes clear in the comparison between survey findings of the Dignity in Care survey and other empirical data is that the data can be understood both within a philosophical framework such as that of Nordenfelt [28,29] and from the microsociological perspective of writers such as Woolhead et al [39]. What is also clear is that findings from the survey replicate findings from previous empirical studies and echo earlier examples of political rhetoric. Frank Dobson (at that time Secretary of State for Health in the UK Government) in 1998, for example said that "no older person in hospital should go without the fundamental care that contributes to recovery – to be helped to eat and drink; to lie in a clean dry bed and to be treated with respect..." The Health Advisory Service 2000 [45] had pointed to deficits in relation to dignity and privacy for older people in acute wards and good practice guidance was subsequently published (Dignity on the Ward; Promoting Excellence in Care) supporting themes discussed in this paper. These remarks sound very like the recent statements from the Department of Health and from current and recent Ministers and Civil Servants, almost ten years later.

## 7. Operationalising Dignity – Implications for everyday practice

We acknowledge the problems of reaching any definitive philosophical account of the concept of dignity. It is probably not possible to develop a set of necessary and sufficient conditions or an account of the essentials of human dignity. Nevertheless we would argue that it remains a useful concept within its own limitations. A minimal account would suggest that it draws attention to a kind of value or worth that is part of our normative account that should shape our relations with and our treatment of other people. At the very least, the concept of dignity calls for an acknowledgement of worth and a concomitant expectation that we should treat people appropriately, with respect for their worth as people.

The empirical data can be read as supporting this position. The concerns expressed by patients and health professionals draw attention to situations in which people felt that they had or had not been treated as being of worth and had or had not been shown appropriate respect for their dignity. The interest in the detailed analysis of patients' construction of their identity and their accounts of their experience comes in the way they choose to exemplify the kind of treatment that does or does not count as dignified in their view. These range from straightforward neglect, as when a patient is left in a soiled bed, to being given proper opportunities for engagement and full participation in decisions about care. It is not surprising that much of what is described as contributing to dignity in care could be grouped under the heading of individualised care.

To operationalise dignity in everyday practice nurses should focus on the four themes discussed in this paper (environment of care; staff attitudes and behaviour; culture of care; and specific care activities). What is also required is the exercise of practical wisdom on the part of policy makers, managers and practitioners. This will enable them to tolerate uncertainty and ambiguity in individual perceptions. There is, for example, a potential conflict between autonomy and dignity. The conflict between the putative right of a French Dwarf to be thrown around a nightclub and a view of the public interest as prohibiting such activities even when freely chosen is perhaps an extreme example [23]. However, if an older person chooses to ignore conventional standards of hygiene and resists attempts to persuade him to have a bath, a consideration of his best interests will have to balance the value of his autonomy and independence against some account of the dangers of self neglect, the distress or offence caused to others and his human dignity. It is interesting to note, for example, that the powers that exist in the UK to remove someone living in unsanitary conditions from his home under the National Assistance Act (1948) require that the removal must be necessary to prevent injury to the health of others or to prevent a serious nuisance to other people: the removal is to protect others rather than to protect the individual concerned. Although the Act is rarely used, this would suggest that the sensibilities of others can be held to be valid grounds for over-riding the autonomy of an individual, whose actions or behaviour may be thought to lack dignity, given sufficient risk or nuisance to others.

## 8. Conclusion

Macklin pointed out that "appeals to human dignity populate the landscape of medical ethics" [35]. Dignity cannot be compartmentalised as but one component of nursing ethics but, rather, is inextricably connected with all of nursing practice. All that nurses do and that nursing aspires to is concerned with promoting, preserving and engaging with human worth or value. It could be argued that the recognition of the worth of others is the only necessary grounds for the existence and maintenance of a nursing service in any society. That something is acknowledged to be of worth and is in some danger of failing to flourish is what provides us with the starting point for nursing. Nursing means to nourish or nurture and to nurse something or someone is by definition to recognise and respond to claims of worth.

Ten years have passed since Frank Dobson made his remarks about the requisite quality of care for older people. We could perhaps have also referred to Barabara Robb's reports published forty years ago, in 1967, under the title of "Sans Everything" [46]. The point is that in spite of the time that has elapsed the problem of dignity in care seems if anything to have become more severe. While it is tempting to speculate, our analysis has not been directed at the determination of causes. We can suggest, however, that our four themes might help to pose fruitful questions for further research. To give brief examples for each in turn:

### • environment of care

The physical environment of care is topical in the UK at the time of writing, as the debate continues about the provision of single- or mixed-sex accommodation. To many people, to judge from comments in the media, sharing hospital wards with people of the opposite sex, when all concerned are unwell, would seem to be a grave affront to dignity. Janet Street Porter [47], a UK newspaper columnist, wrote movingly of her dying sister "enduring the indignity of being placed on a mixed ward, attached to an oxygen cylinder, unable to escape the attention of a naked man masturbating at the end of her bed" . Outside of Intensive Care and Coronary Care Units single sex wards used to be the norm throughout the NHS. A current government health minister has recently described them as an aspiration that cannot be achieved. Lord Darzi [48], responding to questions in the House of Lords, said that "medicine has moved on and, as it has, the design of wards in the health service is based on the disciplines, expertise and competencies of the staff working in those wards... Transforming a ward into a single-sex ward is not achievable. That aspiration cannot be met" . Discussion continues regarding mixed-sex accommodation. The English Health Secretary, Alan Johnson, recently appeared to be modifying the Government's position [49]. This and other issues about the design and building of in-patient accommodation offer rich areas for research, in terms of health policy, hospital architecture and of professional practice.

### • staff attitudes and behaviour

There have been significant changes over the last 20 years in terms of nurse education, staffing levels and skill mix. The increased dependency on greater numbers of unqualified staff or staff with lower qualifications to support reduced numbers of registered nurses, difficulties of recruitment, reliance on overseas recruitment, the use of temporary staff and general changes in society all represent challenges to establishing and maintaining appropriate attitudes and behaviour among nursing staff. Particular problems arise in the care of older people. Management attitudes in the 1980s and 1990s, when there was a concerted drive to separate what were thought by managers to be unskilled tasks such as care related to hygiene and elimination, which did not require a qualified nurse, from those more technical tasks that did, may have contributed to the problem. There is evidence of the relationship between skill mix and the quality of care, but specific work is required to investigate the particular problems of undignified practice.

### • culture of care

The culture of care is complex and is influenced by many factors, some of which will overlap with other issues, such as attitudes and behaviours of staff. The culture of care is also in part a product of the wider institutional culture and this in turn is influenced by government agendas. A popular belief is that government targets have had perverse effects on the way care is managed, for example when pressure to meet targets for maximum waiting times in the emergency department results in patients being moved inappropriately from ward to ward, placed in mixed sex accommodation, or discharged prematurely. Studies that explored the impact and unintended consequences of a target-driven culture might illuminate problems such as these.

### • specific care activities

The performance of specific care activities, such as bathing, toileting, feeding and so on, brings together and could be seen perhaps as the expression of the other three themes. The ability to provide dignified care for the highly dependent patient will be affected by the physical environment, by the attitudes of the staff and by the prevailing culture. Studies that took such activities as a focus and explored the conditions and circumstances that resulted in good or poor practice could help us to understand how complex circumstances interact to impact on a very direct way on the patient experience of care.

There are still gaps in our understanding of dignity, for example, in relation to different perspectives of people in different cultural groups, but enough is known to focus on operationalising respect for dignity in nursing practice. This will require resources for research, education and for action-oriented practice development activities that make a difference to the dignity of patients and staff. Although dignity has been identified as a complex phenomenon, promoting it in everyday practice is neither mysterious nor unachievable. Operationalising dignity requires Government investment and professional will to commend and reward dignity-promoting practice and to respond speedily and constructively to those practices and behaviours that diminish dignity.

## Competing interests

The authors declare that they have no competing interests.

## Authors' contributions

AG led the development of the paper, undertook review of theoretical literature, completed drafts, circulated to the team and incorporated comments. SL reviewed the literature relating to empirical research. PW read and contributed to the development of drafts of the paper. IRJ and DL read and commented on drafts of the paper.

## Pre-publication history

The pre-publication history for this paper can be accessed here:


